# Supplementary material for: Are carbon emissions trading and green financial instruments synergistic? -Comprehensive quantitative research based on content analysis
Source: PLoS One. 2024 Mar 7;19(3):e0298601. doi: 10.1371/journal.pone.0298601 (PMC10919601; doi:10.1371/journal.pone.0298601)
Supplement: S1 Data — (ZIP) [file pone.0298601.s001.zip › Data/地级市Z_F工作报告.zip!/╣π╢1⁄2/╢1⁄2▌╕╩╨/└┤╫╘╣1⁄2╓┌║┼í╛┬φ┐╦╩2╛▌═°í┐_2008.docx]

各位代表：

　　现在，我代表市人民政府向大会作政府工作报告，请予审议，并请政协各位委员和其他列席人员提出意见。

2007年工作回顾

　　过去一年，在省委、省政府和市委的坚强领导下，我们坚持以邓小平理论和“三个代表”重要思想为指导，深入贯彻落实科学发展观，大力推进经济社会双转型，开拓创新，团结奋进，圆满完成了市第十四届人大一次会议确定的年度目标和任务，经济社会发展呈现出“平稳转型、好中求快、安定和谐”的良好局面。

　　过去一年，是我市实施双转型战略的起步之年。我们在深化认识中坚定了转型决心，在学习借鉴中拓宽了转型思路，为推动转型发展打下了扎实的思想基础。我们在强力整治中维护了安全稳定，在完善机制中规范了各项管理，朝着社会转型目标迈出了坚实的一步。我们在外向带动中提升了利用外资水平，在引导扶持中促进了民营经济发展，确保了全市经济在高基数上的平稳较快增长。我们在结构调整中逐步转变了发展方式，在自主创新中逐步增强了发展动力，推动全市经济逐步走上了科学发展的轨道。我们在优化环境中推进了城乡建设，在扶持镇村中促进了区域协调，使城乡一体的发展格局进一步优化提升。我们在改善民生中落实了执政为民，在发展社会事业中推动了全面进步，使广大人民群众更好地分享到经济社会发展的成果。

　　过去一年，是我市综合实力稳步增强之年。经济实力稳步增强。初步预计，全市生产总值3151亿元，比上年增长18.1%；工业总产值6650亿元，增长18.4%；来源于东莞的财政总收入539.5亿元，增长32.7%，其中市财政一般预算收入186亿元，增长44.6%；进出口总额1050亿美元，增长24.7%，其中出口590亿美元，增长24.5%；固定资产总投资840亿元，增长19.1%；社会消费品零售总额695亿元，增长19.0%；金融机构本外币各项存款余额3755亿元，增长8.4%；城市居民人均可支配收入27025元，增长12.2%；农民人均纯收入11514元，增长8%；三次产业比例从0.5：58.1：41.4调整为0.4：57.1：42.5。镇村实力稳步增强。32个镇街可支配财政收入总额159亿元，增长16.1%，平均每个镇街4.97亿元；村组两级可支配收入总额183亿元，增长10.2%，平均每个村（社区）3078万元；村组两级集体资产总值1041亿元，净资产804.5亿元，分别增长7.0%和8.1%，两级集体资产总量约占全省的38%。科教文实力稳步增强。散裂中子源项目落户东莞，全市拥有高新技术企业、名标名牌和专利授权量居全省第四位，我市成为首批省部产学研合作示范市。教育强镇实现全覆盖，各类教育水平实现新提升，高等教育毛入学率60.9%，每万户籍人口升大学88人，在全省地级以上市中名列第一。“图书馆之城”、“博物馆之城”和“广场文化之城”建设初见成效，文化氛围日益浓厚，文明素质日益提高。

　　过去一年，是我市总体形象有效提升之年。我们在全省乃至全国率先启动了农（居）民与职工基本养老保险的统筹发展，率先实现了城乡义务教育全免费，率先铺开了覆盖城乡的污水处理工程建设，率先构筑了高标准的城乡水利防灾减灾体系，率先推行了城乡环卫作业市场化、监理制的管理模式。我们深入推进了全国文明城市、国家环保模范城市、国家园林城市、广东省林业生态市等创建活动，大力开展了城市公共安全八大系列整治，积极探索了特殊城市形态下上千万人口的和谐共建，传承光大了东莞“海纳百川、厚德务实”的城市精神。过去一年，我市社会治安、教育文化、产学研合作、园林绿化、环境卫生、闲置地清理、水利防灾减灾、基本医疗保障、“六好”平安和谐社区创建、产品质量和食品安全等多项工作，得到了上级领导的充分肯定和社会各界的积极评价。东莞先后获得中国优秀创新型城市、中国最具投资价值新锐金融生态城市、广东省社会治安综合治理工作优秀市等荣誉，连续第五次被评为全国科技进步先进城市，全国双拥模范城实现六连冠。在中科院《中国城市发展报告》中，东莞科学发展综合评价位居全国地级以上大城市第二名。在省社科院《2007年广东区域综合竞争力评估分析报告》中，东莞综合竞争力名列全省首位。全市安定和谐的社会形象、创新活力的经济形象、环境优美的城市形象、文明崇德的素质形象，得到了有力提升。

　　回顾过去一年，我们重点抓了以下十项工作：

　　一、强力维护安全稳定。坚持把安全稳定作为社会管理的突出任务，全面铺开了八大系列整治。社会治安方面，深化警务机制改革，推动警力下沉，全市基层一线警力占总警力的88%；推进科技强警，严打违法犯罪，奖励举报违法犯罪和见义勇为人员，全市刑事立案和“两抢一盗”案件同比分别下降5%和8%，治安形势持续好转。“治摩禁电”方面，禁止摩托车营运和电动自行车上路，实行本地摩托车分路段分时段禁行，报废和迁出摩托车8.4万辆，帮助3.5万名“摩的”司机实现转型就业。消防安全方面，对重大危险源和人员密集场所进行全面排查，普查单位达45.4万个，突出开展“三小”场所、出租屋安全隐患整治。建筑安全方面，对1895座桥梁、3134栋旧工业厂房和公用商用建筑进行了检测排查，整治清除了一批隐患。产品质量与食品安全方面，建立完善进货台帐和索证索票制度，大力打击制售假冒伪劣等违法行为，检查生产经营单位16.5万家，查处违法案件1万多宗，取缔无证照经营1.3万家。医疗卫生方面，取缔非法行医窝点1428间次，查处违规医疗机构489间次，“黑诊所”基本清除。水利防灾减灾方面，总投资17亿元的第一批208项工程已动工166项，完工79项；启动了总投资11亿元的第二批158项工程。信访调处方面，建立健全市、镇、村、组（企业）四级信访调解网络，落实21项民生信访问题专项治理，全市共调处民间纠纷1.4万件，成功率达95%；劳动争议案件3.4万件，调解率达92%；群众来信来访下降15%，到省集体上访批次和人次分别下降39%和59%。

　　二、继续加强招商引资。坚持把招商引资作为经济发展的重要措施，不断巩固外源型经济优势。加强园区带动。松山湖协议引资36项130亿元，一批科研院所和企业总部成功落户。虎门港首个大型深水泊位码头竣工投产，协议引资136亿元。东部工业园正式授牌成为省级工业园区。基本完成东部快速路两侧35平方公里土地的统筹，东莞生态园的规划建设开始启动。加强服务保障。认真执行土地利用年度计划与指标统筹制度，优先保障重大投资项目、重点工程和基础设施用地。争取省增加建设用地指标5000亩、用电指标55万千瓦，补贴7900多万元支持地方电厂发电，增加供电能力170万千瓦，有效缓解了资源能源供应紧张问题。坚持和完善外商服务日等制度，推进“大通关”和智能化通关建设，帮助外企解决实际困难。加强宣传推介。积极应对国家加工贸易政策调整，加强政策宣传，消除企业疑虑，增强投资信心。实施以商引商、登门招商和联合招商，组团赴9个国家和地区开展招商推介。全市新签及增资外商投资项目1663宗，合同利用外资31亿美元，实际利用外资21亿美元，分别增长25%和16.1%。

　　三、扎实推进自主创新。坚持以引进消化吸收再创新为基本模式，努力建设创新型城市。强化政策引导。出台“科技东莞”工程11项配套政策，市财政专项支出3.9亿元，扶持科技创新与“两自”企业。全市新增省级高新技术企业67家、省市级民营科技企业255家、省级以上名标名牌104个。强化平台推动。松山湖成为国家火炬创新创业园和广东省知识产权试点园区，东莞华中科技大学制造工程研究院等公共创新平台建设加快。全市拥有国家和省火炬计划特色产业基地9个，专业镇技术创新平台8个，省市级企业工程技术研发中心67个。强化产学研合作。我市被列为国家知识产权试点城市，获批省部产学研结合战略联盟3个、示范基地10个。全市申请专利13000件、授权专利6700件，分别增长32%和36%。全年引进各类人才13万人。

　　四、积极发展民营经济。坚持把民营经济作为自主发展的重要力量，切实加大引导扶持力度。突出政策落实。完善民营经济“新48条”的相关配套政策，用好“创业东莞”工程专项资金、中小企业发展专项资金等，金融机构为本市民企提供超过170亿元贷款。突出扶优扶强。制定“50强民营企业”、“百家成长型民营企业”扶持计划，大力实施“111工程”，提升企业经营管理水平。鼓励支持民营企业上市，培育拟上市企业56家。突出集群发展。加强5个省级产业集群升级示范区建设，设立产业集群发展专项资金，成功举办各类展会72场。全市新增民营单位6.2万户，总数首次超过40万户；民营经济实现增加值993亿元，增长19%，占全市生产总值的31.5%；缴税总额182.6亿元，增长41.2%，占全市总税收的38.8%。

　　五、统筹优化城乡环境。坚持把“五整治”和基础设施工程作为有力抓手，进一步改善城乡环境。扎实推进城市基础设施建设。近两年动工、总投资107亿元的23项市属重点工程已完工14项。投资18亿元，建成投产110千伏及以上输变电工程15项，新增主变容量330万千伏安。完成385公里的LNG管道建设，基本完成市第六水厂一期主体工程。东江航道疏浚整治、小型水库达标建设成效明显。基本完成环境卫生和农贸市场整治。累计投入10.4亿元改善农村卫生条件，建立健全了市镇村三级环卫管理机构，新建改造了一批环卫设施，环卫保洁实现市场化的村（社区）达65%以上；全市586个农贸市场整改达标。深入开展旧村整治和生态环境整治。45个试点村（社区）全面完成，第二批174个村（社区）全面铺开；完成生态整治工程716项。全市新增各类公园、景点、广场237个，新增绿地36平方公里。全市森林覆盖率35.5%，人均公共绿地15平方米。创建国家园林城市和广东省林业生态市分别通过国检和省检。

　　六、有效推动环保节约。坚持把环保节约作为持续发展的重要途径，大力实施“碧水、蓝天、绿地、宜居、绿色GDP”等五大工程。加快环保设施建设。污水处理主体工程动工16项，基本建成8项；配套主干管网工程动工28项，基本建成8项。全面开展污染整治。加强对重点污染企业的排放监控，建立污染举报有奖制度，推行排污限期削减制度，关闭“四纯两小”企业56家。全面完成沙角电厂群脱硫工程，对大型工业锅炉实施烟尘净化改造，对186家冒黑烟企业责令限期整改。严格项目审批，拒批污染项目1231宗。坚决落实节能降耗。促进节约集约用地，累计处置闲置土地4.3万亩，征收土地闲置费3.9亿元。积极开展“双千节能行动”和循环经济试点，促进清洁生产和能源综合利用。全市单位GDP耗地下降15%；前三季度单位规模以上工业增加值能耗和单位GDP电耗分别下降9.45%和5.84%；全市户籍人口自然增长率5.98‰，低于省下达指标。

　　七、大力扶持镇村发展。坚持把扶持镇村作为区域协调的有效途径，统筹城乡发展。加大财政扶持力度。完善镇街工商税收激励机制，并适当向欠发达镇倾斜。将教育经费、最低生活保障金中由村承担的部分，改由市镇两级分担。市财政为每个欠发达村补助10万元办公经费，为欠发达镇贴息1.9亿元、免息借款3.1亿元，并帮助获取贷款34.8亿元。11个欠发达镇各项税收总额52.6亿元，增长28.8%；本级可支配财政收入33.2亿元，增长23.9%，增幅高于全市7.8个百分点。84个欠发达村净资产24.5亿元，增长14.3%，高于全市6.2个百分点，资产负债率下降4.5个百分点。加强农村基础设施建设。市镇总投资20亿元，启动34条共208公里镇街联网公路的升级改造。加强农村集体资产管理。认真开展“八镇百村”示范工程，完成第三届农村干部任期经济责任审计。加快现代农业发展。首批6个市级农业产业园建设全面启动，第二批4个完成规划。新增2家省级和4家市级农业龙头企业。

　　八、全面发展社会事业。坚持社会建设与经济建设同步推进，繁荣各项社会事业。优先发展教育事业。市财政增加投入9.2亿元，推动三级办学向两级办学转变，推进高中联合办学、规模化办学，启动了市职业技术学院建设。繁荣发展文体事业。全面铺开公益性文化事业单位改革，继续开展公益文化活动招商，成交项目80个，签约金额7500万元。加强文化市场综合执法，“黑网吧”整治收效明显。成功举办首届东莞国际音乐剧节和第三届东莞读书节。有线电视数字化整体平移工程进展顺利。公共体育设施加快完善，全民健身活动广泛开展，竞技体育水平提升，莞籍运动员获得国际赛事金牌3枚、全国赛事金牌25枚。协调发展卫生社保等各项事业。启动了总投资25亿元的“四院一中心”建设，大力推进基层和社区医疗服务网点建设，全市新增基层和社区医疗机构454家。推动社会保障扩面发展，在全省率先将建筑企业施工作业人员全面纳入工伤保障范围，全市各险种参保人数增长30.6%，净增328.3万人次。加大财政补贴，提高农（居）民基本医疗保险待遇，建立地方养老保险与企业年金制度，调高了城乡最低生活保障标准，完善了社会救助体系。启动廉租房和经济适用房建设。全市公园和公厕全面实行免费。积极帮扶西藏林芝、广西河池、广东乳源等地区。民族宗教、外事侨务、工青妇幼、科学普及、档案方志和国防、气象、统计、打私、老龄等工作也扎实推进。

　　九、认真办好十件实事。坚持把改善民生作为构建和谐的基础工程，承诺为群众办好的十件实事基本完成。一是增加了视频监控点，建成社区警务室500个、各类治安监控摄像头7.8万个。二是加强了农产品供应保障，建成市农业检测检验中心，建立异地供莞生猪定点基地51个、无公害水产品生产基地3000亩。三是健全了社区医疗卫生服务网点，建成社区卫生服务中心60个，社区卫生服务站190个。四是优化了公共交通，投入10.2亿元，新增公交线路148条、公交车辆1301辆，投放“公共的士”1500辆，更新出租车900辆，开通“一元”公交线路32条，新建公交站亭一批。五是推进了污水处理工程建设，主体工程和配套主干管网工程分批次抓紧实施。六是完善了公共环卫设施，新建改建公厕907座、垃圾转运站512座，整改规范垃圾填埋场。七是减轻了教育负担，对8294名“低保”家庭子女发放助学金，对2.2万人次实施助学减免。八是改善了农村通路通水状况，投入2.8亿元，建成水泥公路191公里，供水管网253公里。九是促进了就业和残疾人帮扶，资助培训户籍人员1.98万人次，免费推荐就业2.15万人次，新增就业岗位14.2万个，城镇登记失业率1.98%，为残疾人提供专项补助、康复医疗和就业培训2万多人次。十是维护了新莞人权益，培训新莞人18.3万人次，1.9万家企业纳入工资监控，为19.2万名员工追回欠薪2.2亿元，接收43.7万名新莞人子女在我市义务教育阶段学校就读。

　　十、深入推进依法行政。坚持以提升政府施政水平为目标，全面推进依法行政。着力规范政府行为。加强规范性文件管理，完成47个行政执法部门的职权界定，认真做好行政复议和应诉工作，健全了政府法律顾问、专家咨询论证等制度，对全市行政执法人员实行培训考核、持证上岗。着力推进综合执法。成立市城市管理综合执法局，整合规划、市政、环保等8个方面共106项具体职能，实行统一执法。着力优化政府服务。深化第三轮行政审批事项改革，进一步削减审批事项，简化办事程序。启动行政审批电子监察系统二、三期建设，全市41个单位共404项行政审批事项部分流程实现网上审批，46个单位和14个镇街设有“一站式”办事窗口。着力推动阳光行政。依法扩大政务公开范围，严格执行政府采购、建筑工程公开招投标和经营性用地、工业用地招拍挂制度，深入开展房地产市场秩序整治和商业贿赂专项治理，扎实开展民主评议行风和“市民评机关”活动。着力加强绩效评价。建立投资失误责任追究制度，规范政府投资行为。对市财政安排200万元以上和跨年度支出项目统一进行绩效评价，推进经济责任审计和效益审计，提高财政资金使用效益。加强市属资产监管，促进保值增值。

　　各位代表！过去一年，我们在科学发展上争先创优，在构建和谐上力求突破，在推动转型上攻坚克难，全市经济社会发展取得了可喜的成绩。这些成绩的取得，离不开上级党委、政府和市委的正确领导，离不开市人大、市政协与各民主党派的大力支持，离不开广大市民以及驻莞部队的共同努力，离不开港澳台同胞、海外侨胞以及国际友人的关心帮助。在此，我代表市政府，向所有参与、支持和关心东莞现代化建设的各界人士，表示衷心的感谢！

　　同时，我们也清醒地认识到，全市经济社会发展还存在不少问题和隐忧，主要表现为：产业结构不够合理，经济发展方式转变任务艰巨，持续稳定发展压力增大，加工贸易转型十分迫切，自主创新能力亟待增强；资源环境问题仍然突出，污染治理与环境保护力度仍需加大；公共安全形势依然严峻，消防隐患大量存在，社会管理任务繁重；消费物价持续上涨，民生保障有待改善，区域协调有待加强，城市文明与社会和谐程度有待提高，等等。对于这些问题，我们将高度重视，采取有力措施逐步加以解决。

2008年工作安排

　　2008年是我市双转型战略逐步转向深入的一年。我市的发展既面临许多机遇，也面临不少挑战。世界经济仍将保持平稳增长，国际产业转移趋势增强，有利于我市扩大开放促发展；国内经济平稳较快，政局和谐稳定，有利于我市抓住机遇促转型。与此同时，原材料价格上涨、人民币汇率变动、国际贸易摩擦等不确定因素增加，国家宏观调控继续加强，加工贸易政策深化调整，区域竞争日益加剧，劳动合同法和新的企业所得税法正式实施，都需要我们积极应对并加快适应；我市转型调整力度不断加大，一定程度上可能会造成增长速度的放缓；2007年房地产、股市、契税收入等非常规的发展，也给相关经济指标的持续增长带来了一定压力。对此，我们必须深刻审视所处环境变化，深刻分析优势与不足，深刻反思思想精神状态，以解放思想转变发展观念，以改革创新破解发展难题，既要增强忧患意识，保持清醒头脑，又要坚定发展信心，勇于攻坚克难，努力在推动转型中实现新的发展。

　　综合考虑各方面因素，今年全市经济社会发展的主要目标确定为：生产总值增长14%；单位生产总值能耗下降4.4%；市财政一般预算收入增长14%；固定资产投资总额增长15%；社会消费品零售总额增长16%；外贸出口总额增长10%；实际利用外资增长10%；居民消费价格指数上升控制在3%左右；城市居民人均可支配收入增长9%；农民人均纯收入增长7%；人口自然增长率控制在6.4‰以内。

　　今年政府工作的指导思想是：高举中国特色社会主义伟大旗帜，以党的十七大精神为指导，以贯彻省委十届二次全会要求为动力，认真落实科学发展观，深入推进经济社会双转型。在继续强化社会管理、深化社会转型的基础上，突出以经济转型为重点，力求在优化经济结构、推动产业升级、培育新的经济增长点上取得明显进展，努力实现经济社会又好又快发展，争当实践科学发展的排头兵。

　　为此，要做到“六个坚持”：坚持解放思想、改革开放，把思想从不适应、不利于科学发展的认识中解放出来，从“见物不见人”的发展观中解放出来，完善体制机制，创新管理手段，扩大对外开放，激发转型发展的活力。坚持好字优先、稳中求进，处理好转型与发展的关系，落实好宏观调控政策，确保经济运行更加稳健；坚持创新驱动、内外协调，进一步优化经济结构，转变经济发展方式，提高经济发展质量；坚持城乡统筹、一体发展，加快基础设施和环境建设，加大扶持镇村力度，全面提升城乡环境和发展水平；坚持集约利用、环保节约，坚决落实环保节能各项要求，加大攻坚力度，确保节能减排取得新的进展；坚持以人为本、执政为民，更加重视民生改善，更加注重和谐构建，加快发展各项社会事业，促进公共服务均等和发展成果共享。

　　具体要抓好以下十个方面的工作：

　　一、突出抓住四个重点，着力优化产业结构

　　产业结构优化是我市经济转型的重中之重，必须抓住关键，重点突破，着力构建现代产业体系。

　　以信息产业为突破做强高新技术产业。以松山湖科技产业园为载体，建立信息产品研发区、创建信息学院、设立软件产业外包平台等，通过完善配套及相关优惠，吸引IT企业集中设立研发机构和企业总部，集聚研发人才。推动省市联手共建电子信息及电气机械产业升级工程，用好扶持政策和专项资金，突出支持重点企业、重点技术、重点品牌和重点平台的建设。加快发展生物制药、光机电一体化、新材料和新能源、新型平板显示等高新技术产业。

　　以产业集群为依托提升传统产业。大力推进五金模具、服装、家具、毛织等省级产业集群升级示范区建设，培育发展新兴产业集群，积极申报第三批省级产业集群示范区。抓好一批行业性公共服务平台建设，为产业集群提供技术研发、质量检测、信息咨询等基础性服务。积极采用先进适用和关键技术，大力改造提升传统产业。推广专业镇、专业市场与会展业联动发展的模式，增强市场辐射力。逐步淘汰一批高污染、高耗能、低效益企业，推进产业转移园区的建设。

　　以商贸东莞为抓手繁荣服务产业。大力发展现代服务业，鼓励发展会计、法律、资产评估、科技咨询、教育培训等生产性服务业，稳步发展工业设计、广告策划等创意产业；加快建设金融强市，推动地方金融创新，发展利用资本市场。大力发展商贸流通产业，培育商贸龙头企业，壮大会展、旅游、文化等产业；研究制定各镇街中心区“退二进三”的配套政策；结合建市20周年等重大活动，加大城市营销力度，提升东莞对外形象。

　　以企业培育为重点壮大民营经济。深入落实民营经济“新48条”，采取有效措施，促进资产盘活，着力解决民营企业融资难、引智难、办证难等问题。重点扶持民营企业50强、百家成长型企业。落实培育企业上市工作方案，做好各项服务，力争民营企业上市实现突破。

　　二、实施三大外经工程，提升外源经济质量

　　外源型经济是我市的特色和优势，必须立足巩固，着力提升，质量并重，推进转型升级。

　　大力实施加工贸易转型工程。积极适应国家加工贸易政策调整，突出产品内销和企业研发两大重点，推动加工贸易从单纯加工制造逐步向研发、销售环节延伸。加强外资企业转型升级辅导，及时向企业宣讲国家最新政策法规。探索设立奖励基金，构筑促进转型的服务中心和信息平台，组建口岸保税物流中心，建立企业内销“快速通道”，为加工贸易企业转型升级提供全方位的支持服务。

　　大力实施关键项目引进工程。重点引进产业链缺失项目，强化产业配套优势；重点引进大型项目，强化项目带动能力；重点引进生产性服务项目，强化生产服务支撑。重点保障关键项目用地需求，完善跟踪服务，及时解决关键项目在选址投产等方面遇到的问题。

　　大力实施监管模式再造工程。创新加工贸易业务电子审批模式，完善镇街电子审批分平台，加快审批系统升级，逐步实现全面覆盖。创新加工贸易监管模式，推动计划合同审批向实际合同审批转变，做好全省加工贸易联网管理试点工作，逐步实现企业、外经贸和海关的三方联网。加快东莞电子口岸平台建设，整合外经贸、海关、外汇、税务、检验检疫等部门信息资源，实现同一平台的电子化管理。

　　三、加快园区开发建设，强化龙头引领作用

　　园区是我市转型发展的重要载体和示范区域，必须清晰定位，完善配套，加快建设，发挥引领转型的龙头作用。

　　加快强化松山湖科技产业园的功能。以国家火炬创新创业园的建设为契机，进一步优化园区布局，增强松山湖作为全市科技中心的功能。加快重大工业项目的投产步伐，早日形成规模效应。加快东莞中子科学中心等科研院所建设，做好与各镇街企业对接工作，增强科技辐射带动能力。启动生物信息港、创意设计园等项目建设，探索建立公司化运营体制，有效聚集研发企业和研发人才。推进滨湖生态旅游区建设，增强园区环境优势。

　　加快提升虎门港的建设水平。实施港城互动、产业带动，加快基础设施建设，完善港口功能配套。完成沙田港区5号、6号泊位项目建设，启动7号、8号泊位等项目建设。全面启动三大作业区二期市政配套工程，推进保税物流中心（B型）和长安港区建设，加快立沙岛整体开发步伐，使虎门港成为带动西部地区及沿海产业发展的龙头。

　　加快推进东莞生态园和东部工业园的开发。按照湿地生态园、高端产业发展及配套服务区的定位，编制完善东莞生态园的相关规划，启动水系综合整治和生态绿化建设，启动园区填土工程及生态园大道等10项道路工程，抓紧市高技能实训基地等项目动工。加大东部工业园统筹协调力度，加快开发建设和招商引资步伐。

　　加快整合镇街工业集聚区。完善区内基础设施以及商业、文化、医疗卫生等功能配套，改善厂区及周边环境。结合旧村、旧厂改造，整合土地资源，优化厂区布局。创新管理服务，加强招商引资，大力发展特色产业，逐步提升产业层次。

　　四、完善科技创新体系，增强自主创新能力

　　科技创新是推动转型发展的根本动力，必须落实创新政策，扶持创新企业，打造创新平台，全面增强自主创新能力。

　　积极落实科技政策。认真落实“科技东莞”工程第一批11项配套政策，尽快制定出台第二批配套政策，用好10亿元的专项资金及各镇街的配套资金。以实施国家《科技型中小企业创业投资引导基金管理办法》为契机，探索组建我市科技风险创业投资机构，为科技型中小企业提供资金支持。

　　积极培育“两自”企业。帮助科技型龙头企业争取863项目、火炬计划项目、粤港关键领域技术突破招标等重大科技专项，开展关键技术攻关。加大知识产权保护力度，鼓励企业申请自主技术专利、研制技术标准。深入实施质量兴市和名牌带动战略，加快产品质量检测基地建设，加强产品质量管理，鼓励企业争创名牌，推动区域品牌创建。

　　积极加快平台建设。加快建设莞港生产力促进基地等公共技术平台，加快建设留学人员创业园、博士创业园等孵化平台，加快建设国家火炬计划特色产业基地、专业镇等行业创新平台，加快建设一批国家、省、市级企业技术中心。按照“政府资助、市场运作”的原则，加强平台运营管理，充分发挥平台功能。

　　积极推进产学研合作。以实施产学研结合示范市工程为契机，分行业、分专题组织企业科技合作对接活动，组建一批产学研合作战略联盟，大力推进高校、科研院所与企业、行业的合作，推动创新成果转化。拓宽对外科技合作地域领域，重点加强与香港等地区的交流合作。

　　五、加强公共安全管理，确保安全稳定有序

　　公共安全管理是社会稳定发展的重要保障，必须深入开展专项整治，健全长效管理机制，全面提高应急能力，有效维护安全稳定有序局面。

　　深入推进专项整治。继续狠抓社会治安，加强公安“三基”工程建设，充实基层警力，突出整治治安重点部位，重拳打击暴力犯罪、黑恶势力犯罪以及“两抢一盗”、公交扒窃、驾车抢劫等严重违法犯罪，促进治安持续好转。继续狠抓消防安全，落实安全责任，完善安全设施，强化基层基础，加大执法力度，对重大危险源、“三小”场所、出租屋及其他人员密集场所等进行大排查、大整改，对业主、经营者及从业人员进行安全知识大培训，严防重特大事故发生。继续狠抓信访调处，深入贯彻劳动合同法，加强劳动监察，落实领导包案，妥善处理劳资纠纷、土地信访等突出问题。继续狠抓“治摩治吧”、建筑安全、食品药品安全、医疗市场、防灾减灾等专项整治。

　　着力加强长效管理。坚持完善机制，深入开展治安“端窝”、“断腿”、“清源”行动，设立新莞人服务管理局，加强出租屋及流动人口管理，建立健全各类公共安全管理机制。坚持依靠科技，完成第二批全国科技强警示范城市建设工作，推进110、119、122三台合一，推动视频监控进社区、进出租屋、进公交车。坚持群防群治，广泛动员群众参与公共安全管理，鼓励见义勇为，研究建立安全隐患有奖举报制度，加强宣传教育，提高群众安全意识和自救技能。

　　全面提高应急能力。落实应急管理的机构、人员、经费和物资储备，编制和完善各级各类应急预案，发挥公安、消防、武警、解放军和预备役民兵的骨干作用，建立专业应急救援队伍，加强应急演练。健全监测预警系统，加强社区、农村、学校、医院、企事业单位等基层组织的应急管理，做好突发公共事件预防和应对工作。

　　六、统筹城乡一体发展，促进区域协调平衡

　　坚持以城带乡，以工促农，统筹城乡建设，大力扶持镇村，全面提升城乡环境建设和经济发展水平。

　　加快推进城乡基础建设。贯彻城乡规划法，启动全市总体规划修编，做好控制性详细规划的编制和实施，强化规划控制管理。探索建立合理公开的征地拆迁补偿机制。推进轨道交通立项审批，抓紧市属重点工程建设，争取完成人民医院新院、莞深高速三期等15项。加快推进镇街联网路升级改造工程，完成一期34项，启动二期10项。加快城乡水利防灾减灾工程建设，全面完成第一批208项，基本完成第二批158项。抓紧莞长C线供水工程，完成第六水厂一期优质水工程。建设110千伏及以上输变电工程49项，力争年内投产23项，新增主变容量406万千伏安；加快镇街天然气储配站及管网建设。

　　深入实施农村环境“五整治”。全面落实环境卫生和农贸市场整治的后续工作。基本完成生态环境整治，市镇生态建设工程年内要完成90%以上的工程量。在所有村（社区）全面铺开旧村整治。推进农民公寓的规划建设和旧村、旧厂区的整合改造。大力整治农村“六乱”现象。

　　加力扶持镇村转型发展。继续落实税收返还、贴息贷款等政策，强化引导激励，推动镇村特别是欠发达镇村转型发展。加大财政转移支付，研究制定土地转让收入部分返还被征地农村集体等机制，进一步减轻基层负担。做好第四届村（居）委会选举工作，推进“五有”新村、“六好”平安和谐社区建设，稳妥实施“村改居”。推进农业产业园和农田标准化建设，扶持培育农业龙头企业，发展特色农业、观光农业和海洋渔业。

　　深化农村集体资产管理。落实农村重大事项审查制度，强化农村审计监督，开展清资减债工作，堵塞各种管理漏洞，防止超前分配和盲目投资行为，避免基层支出刚性增长。加强农村集体经济转型问题研究，开展村级统一核算试点，创新集体经济经营发展模式，确保农村集体资产保值增值。

　　七、坚持环保节能减排，推进生态文明建设

　　环保节约是科学发展的内在要求，必须强化环境综合治理，集约节约利用资源，深入创建国家环保模范城市，努力打造生态宜居城市。

　　推进环保设施建设。基本完成36项污水处理工程和配套截污主干管网工程建设，启动支次管网工程建设，进一步提高全市生活污水集中处理率，努力实现污水处理城乡全覆盖。加快医疗废物处理中心、潲水集中处理中心和3座生活垃圾处理厂的建设，动工兴建淤泥处理工程。

　　推进运河综合治理。按照“截污、清淤、活源、治堤”的总体思路，对运河水系进行全方位的整治，范围包括石马河、东引运河、寒溪水流域以及东莞生态园水系和各镇街内河涌。年内要启动流域清淤、补水活源、面源清理、堤岸建设及景观整治等工程。

　　推进污染源头防控。严格执行生态绿线规划，强化生态保护和水源保护。加强对重点污染企业的监管，督促255家企业落实污染削减措施，对301家企业实行在线监测，对238家“四纯两小”企业实施关闭。加强大气和噪声污染治理，推进锅炉烟尘净化改造，开展机动车尾气污染整治，市财政投入7200万元，资助推动全市公交车、出租车“油改气”。规范畜禽养殖业管理，遏制养殖业污染反弹。积极整治露天垃圾填埋场。

　　推进节能降耗任务落实。加快建立科学的节能降耗统计、监测和考核体系，推进市能源利用监测中心建设，深入开展“双千节能行动”和循环经济试点工作。全面推进建筑节能，加强可再生能源的综合利用。加快土地利用总体规划修编，认真执行国有工业用地招拍挂制度和最低出让价标准，积极消化闲置土地，严格做到依法和集约用地。推进节水节电，倡导绿色消费，建设节约型社会。

　　八、加快发展各项事业，推动社会全面进步

　　社会建设是构建和谐的重要内容，必须加快发展各项社会事业，推动经济社会协调发展。

　　推动教育优质化均衡化发展。深入推进办学体制改革，优化城乡教育结构，推动各级各类教育协调发展。加强师资建设，提升教学水平。动工新建扩建12所高中阶段学校。大力发展职业教育，推动职业高中联合办学，为制造产业发展提供高素质的技术人才支撑。探索新莞人子女在我市接受义务教育的准入条件，力争为新莞人子女提供更多学位。

　　推动文化事业创新发展。积极创建全国文明城市，大力弘扬“海纳百川、厚德务实”的城市精神。深化文化事业管理体制改革，加快现代传媒建设，完善公益文化活动项目招商。加大文物和非物质文化遗产保护力度。完成岭南画院、文学艺术院、可园博物馆建设，加快群众艺术馆新馆建设。大力发展企业文化和社区文化，广泛开展文化节目进社区活动，让基层群众共享文化发展成果。

　　推动卫生体育社保等事业发展。加快“四院一中心”等公共医疗设施建设，按照“政府办政府管”的思路，健全社区卫生服务，打造一刻钟医疗服务圈。完善体育设施，以北京奥运为契机举办系列群众体育活动，打造半小时体育生活圈。继续完善城乡一体、覆盖更广的社保体系，完善预防、保险、康复三位一体的工伤保障，建立失业保险与促进就业联动机制，发展企业年金，抓好企业退休人员社会化管理服务。探索建立社会工作制度，加强对民间组织的引导和管理。积极推进廉租房和经济适用房建设。严格执行国家计划生育政策，提高出生人口素质，稳定低生育水平。继续做好对口帮扶、区域协作、双拥共建、人民防空、民族宗教、外事侨务、港澳台务、气象、统计、档案、方志等工作。

　　九、坚持办好十件实事，有效解决民生问题

　　坚持每年解决一批与民生密切相关的热点难点问题，今年继续为群众办好十件实事：一是创建100个平安社区，新建120个社区警务室。二是建成市区公交站亭站台520个，站场40个，增加公交班次，延长服务时间。三是建成廉租房15000套、经济适用房3300套。四是完成30条、总长100公里的村际联网道路升级改造。五是基本建成36项污水处理厂及配套截污主干管网工程。六是市镇财政安排5.8亿元，整合建设社区卫生服务中心34个、服务站342个。七是市财政安排6000万元，为只参加住院基本医疗保险的企业职工和农（居）民增加门诊医疗保障。八是市财政安排2800万元，为户籍中职学校在读生提供每人1500元助学金；免收义务教育阶段户籍借读生杂费差额。九是市财政安排5400万元，将最低生活保障标准提高到每人每月400元；安排2000万元，为1万名户籍困难残疾人提供生活专项补助、康复服务和技能培训。十是新增就业岗位13万个；市财政安排1.8亿元，资助10.2万人次接受技能培训。

　　十、深化行政效能建设，提高政府施政水平

　　以提高行政效率为主线，加快政府职能转变，提升行政执行力，建设服务型政府。

　　创新管理促提效。健全政府职责体系，落实部门主办责任制和行政执法层级监督协调机制，防止推诿扯皮行为。以推进综合行政执法改革为契机，探索职能有机统一的大部门体制，解决部门职能交叉、多头执法等问题，逐步理顺市直部门派出机构与镇街政府的权责关系。深入推进第三轮行政审批事项的清理和调整，推进网上审批和并联审批，简化办事程序。做好长安镇综合配套改革试点工作。

　　监督考评促提效。建立健全体现科学发展观和正确政绩观要求的干部考核评价体系。强化行政效能监察，推进电子监察系统建设，确保政令畅通，执行到位。推进财政资金绩效评价和审计，提高财政使用效率。贯彻落实《政府信息公开条例》，推行公共企事业单位办事公开制度，加强政府新闻发布工作。积极推进依法行政，落实行政执法责任制和过错责任追究制。坚决执行人大决议，认真听取各方意见，主动接受市人大、市政协及社会各界的监督。

　　改进作风促提效。大兴求真务实之风，深入开展推动转型发展的调查研究，增强政府工作的前瞻性、系统性和主动性。深入落实改进机关作风六项制度，纠正部门和行业不正之风，进一步优化政府服务，提高办事效率。大力精简会议和文件，改进文风会风。深入推进廉政建设和反腐败斗争，建设务实、清廉、为民、高效的政府。

　　各位代表！人民的支持和监督，是我们不断前进的强大动力。让我们同心同德，再接再厉，以坚定的目标激发热情，以高昂的斗志克难奋进，以扎实的工作再铸辉煌，为推动经济社会双转型、建设富强和谐新东莞做出新的更大的贡献！
